# Probiotic Engineering and Targeted Sonoimmuno‐Therapy Augmented by STING Agonist

**DOI:** 10.1002/advs.202201711

**Published:** 2022-05-23

**Authors:** Dan Lu, Liying Wang, Liping Wang, Liwei An, Minfeng Huo, Huixiong Xu, Jianlin Shi

**Affiliations:** ^1^ Department of Medical Ultrasound Shanghai Engineering Research Center of Ultrasound Diagnosis and Treatment National Clinical Research Center of Interventional Medicine Ultrasound Research and Education Institute Shanghai Tenth People's Hospital Shanghai Frontiers Science Center of Nanocatalytic Medicine School of Medicine Tongji University Shanghai 200072 P. R. China; ^2^ State Key Laboratory of High Performance Ceramics and Superfine Microstructure Shanghai Institute of Ceramics Chinese Academy of Sciences Research Unit of Nanocatalytic Medicine in Specific Therapy for Serious Disease Chinese Academy of Medical Sciences (2021RU012) Shanghai 200050 P. R. China

**Keywords:** bifidobacterium, immunotherapy, probiotic engineering, sonodynamic therapy, stimulator of interferon genes agonist

## Abstract

Tumor targeting and effective immunomodulation are of critical significance during tumor treatment by sonodynamic therapy (SDT). Herein, the probiotic engineering of the clinically approved sonosensitizer (hematoporphyrin monomethyl ether (HMME)) is reported onto the probiotic bacterium *Bifidobacteria Longum* (BiL) for sonosensitive bifidobacterium construction (HMME@BiL cells). Based on the hypoxic tropism feature of the strain, effective tumor‐targeted sonodynamic therapeutics can be achieved both in vitro and in vivo. To improve the immunological responses against tumor during sonodynamics, a recently‐developed stimulator of interferon genes immune agonist SR717 has been employed to improve the anti‐tumor immunity with prominent activities, eradicating both primary and metastatic tumors with high efficiency and satisfied biocompatibility. The present work provides a promising paradigm of microbiotic nanomedicine in a sophisticated sonoimmunotherapeutic strategy against malignant tumors.

## Graphical abstract

Engineering of the clinically approved sonosensitizer hematoporphyrin monomethyl ether onto the probiotic bacterium *Bifidobacterial Longum* for effective tumor‐targeted sonodynamic therapeutics has been presented. Synergized with the recently‐developed stimulator of interferon gene immune agonist SR717 stimulated anti‐tumor immunoresponses, comprehensive sonoimmunotherapy with high therapeutic selectivity, efficacy, and biocompatibility has been achieved.

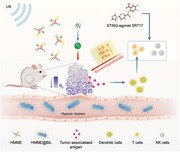

## 1 Introduction

Sonodynamic therapy (SDT) mediated by focused ultrasound (with frequency of 1.0–2.0 MHz) has been one of the most promising non‐invasive physical irradiation tumor therapeutic modalities with high biocompatibility and effectiveness in clinics. During a typical SDT process, low‐frequency ultrasound is employed to activate the tissue‐accumulated sonosensitizers for reactive oxygen species (ROS) generation and subsequent ROS‐mediated tumor destruction.^[^ ^1^ ^]^ With the advantages of deep tissue penetration, non‐invasiveness, and high controllability features, the SDT modality has been one of the most favorable tumor therapeutics among the science community and receiving broad investigations. For sonosensitizers, hematoporphyrin monomethyl ether (HMME) has been one of the clinically‐approved sonosensitizers for practical applications with prominent SDT performance. Nevertheless, due to the low molecular weight (672 Da), the drug bioavailability of HMME has been typically constrained. In addition, vein‐administrated HMME travels to the tumor tissue based on the passive circulation along with the plasma, resulting in limited tumor localization. The present obstacle could eventually lead to mitigated therapeutic effect, further constraining the practical applications.^[^ ^2^ ^]^ During the past years, various nanoplatforms have been designed to improve the bioavailability as well as the tumor‐localization of the sonosensitizers. Typically, researchers have encapsulated the sonosensitizers into the drug delivery system (e.g., liposome,^[^ ^3^ ^]^ hollow mesoporous silica nanospheres,^[^ ^4^ ^]^ titanium dioxide nanoparticle,^[^ ^5^ ^]^ etc.) for passive targeting transportation through enhanced permeability and retention effect. These approaches could not only substantially prolong the plasma half terminal life of the small molecule sonosensitizers, but also impart the stimuli‐responsive characters (e.g., pH‐responsive,^[^ ^6^ ^]^ GSH‐responsive,^[^ ^7^ ^]^ H_2_O_2_ responsive,^[^ ^8^ ^]^ etc.) within the lesion tissue based on the specific microenvironment. Nevertheless, further improvement of the tumor‐accumulation efficiency of the delivery nanoplatforms is still demanding.

The recent decades have witnessed the growing research on the identification of tumor microenvironments, initiated as tumor occurrence and developed as tumor growth.^[^ ^9^ ^]^ Tissue hypoxia is one of the major characteristics of the tumor microenvironment that results from the imbalanced blood supply and cell consumption during proliferation.^[^ ^9a^ ^]^ Inadequate cellular oxygenation regulates the delayed degradation of Hypoxia‐Inducible Factor 1‐Alpha, and transcriptionally modulates a variety of cellular responses including glycolysis, angiogenesis, immunosuppression, ultimately leading to the exaggerated growth, metastasis, and cell‐death resistance of the tumor cells and tissue with adaptation.^[^ ^10^ ^]^ Although tumor hypoxia has endowed prominent resistance against the chemodrugs and radiations, these hallmarks can also offer promising opportunities to design advantageous therapeutic approaches for cancer treatment, including hypoxia‐activated prodrugs (e.g., TH‐302,^[^ ^11^ ^]^ PR‐104A^[^ ^12^ ^]^), hypoxia‐activated nanomedicine (e.g., Albumin‐MnO_2_ particles,^[^ ^13^ ^]^ covalent‐organic framework^[^ ^14^ ^]^) and hypoxia‐responsive gene therapy (e.g., retroviral vector^[^ ^15^ ^]^).

Considering the native tropism toward hypoxia of anaerobic microorganisms, the application of anaerobic bacteria as the drug delivery system for tumor therapy can be highly appealing.^[^ ^16^ ^]^ Bifidobacterium is a genus of Gram‐positive, non‐spore‐forming probiotic bacteria that belong to the Bifidobacteriaceae family of the Actinobacteria phylum. Bifidobacterium Longum (BiL) has been one of the probiotic bacterium that is found to colonize the gastrointestinal tract of infants with microaerotolerant characteristics.^[^ ^17^ ^]^ These microbes have been applied as probiotics for the treatment of diseases such as necrotizing enterocolitis,^[^ ^18^ ^]^ infantile colics,^[^ ^19^ ^]^ and allergies.^[^ ^20^ ^]^ Bifidobacterium has been demonstrated to selectively accumulate in tumors when entering the bloodstream, probably due to the tropism of the hypoxic microenvironment and the immunosuppressive conditions for bacterial colonization and proliferation. It was found that intralesional repeated injection of *B. infantis* inhibited the growth of Meth‐A tumor cells transplanted subcutaneously into BALB/c mice.^[^ ^21^ ^]^ Extensive research has shown that *Bifidobacterium spp*. can improve anticancer immunosurveillance and increase the abundance of tumor‐infiltrating CD8^+^ T cells.^[^ ^22^ ^]^ Therefore, Bifidobacterium has been considered as the promising delivery vector for tumor‐specific transportation.

Recent studies have demonstrated that immunogenic cell death induced by external irradiation such as laser and ultrasound, could stimulate the presentation of the tumor antigens and trigger the innate antitumor immune responses.^[^ ^23^ ^]^ Considering the immunosuppression character of the tumor cells and tissue, inadequate responses could be observed when an independent SDT modality was employed.^[^ ^24^ ^]^ Immunomodulators such as the immune checkpoint blockade (anti‐programmed cell death‐Ligand 1,^[^ ^1^ ^]^ cytotoxic T lymphocyte‐associated protein 4,^[^ ^25^ ^]^ and indoleamine‐pyrrole 2,3‐dioxygenase inhibitors^[^ ^26^ ^]^) have been combined to alter the immune‐microenvironment, facilitating the promising synergistic therapeutics. Cyclic guanosine monophosphate (GMP)‐adenosine monophosphate (AMP) synthase (cGAS)‐stimulator of interferon genes (STING) (cGAS‐STING) signaling pathway plays a critical role in innate response against infection.^[^ ^27^ ^]^ It has been indicated that STING contributes to the intrinsic antitumor immunity by activation of type I interferons and other inflammatory cytokines in recognition of the extracellular non‐self DNA.^[^ ^28^ ^]^ Clinical research on low‐grade serous carcinomas of the ovary, which is known to be genomically stable and immunosuppressive, has been demonstrated to harbor high STING expression,^[^ ^29^ ^]^ implying the perspectives for active agonists against immunosuppressive tumors. SR717, a non‐nucleotide, small‐molecule STING agonist overcoming the limitation of metabolic instability of the natural cyclic dinucleotide (CDN) ligands has been demonstrated to exhibit antitumor activity through promotion of the activation of CD8^+^ T, natural killer, and dendritic cells in relevant tissues, meanwhile facilitating the antigen cross‐priming process during the immune responses.^[^ ^30^ ^]^ Accompanied by the SDT modality, such agonist is believed to generate prominent sonoimmuno‐therapeutic consequences against malignant tumors with high efficiency.

**Scheme 1:**
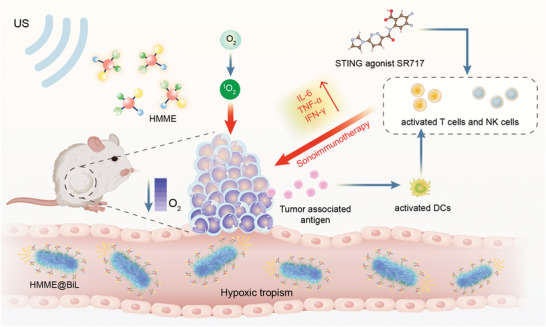
Schematic illustration of probiotic‐microbe‐based SIT against cancer with STING agonist.

In the present work, we reported the chemical engineering of the sonosensitizer HMME onto tumor‐targeting BiL (HMME@BiL) for prominent tumor destruction with high biocompatibility and efficiency (**Scheme** **1**). In addition, recently proposed STING immune agonist SR717 has been employed to stimulate the anti‐tumor immunoresponses via promoting CD8^+^ T cells and nature killer cells (NKs) maturation and cytokine secretion, enabling the sonoimmunotherapy (SIT). We found that combination SIT treatment by HMME@BiL + US + SR717 could not only effectively suppress the primary tumors but also mitigate the progression of tumor metastasis in murine CT26 colorectal cancer metastasis models. This work illustrates the promising applications of highly tumor‐specific SIT enabled by the combined application of microbial engineering and STING agonist for comprehensive tumor therapeutics.

## 2 Culture and Characteristics of *Bifidobacterium Longum* (BiL)

**Figure 1:**
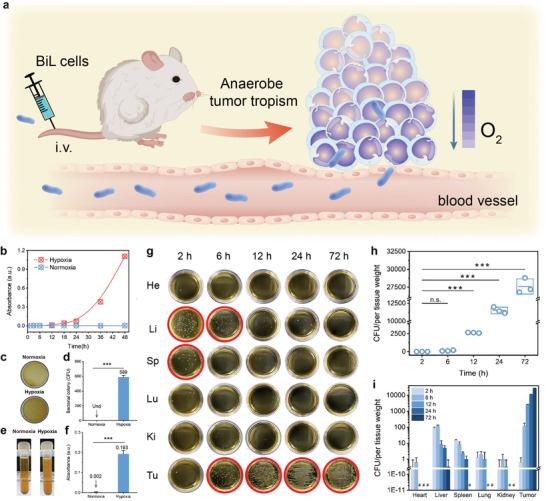
Culture and the anaerobism of the probiotic microbe *Bifidobacterium Longum* (BiL). a) Schematic illustration of BiL‐based tumor tropism. b) Optical densities evolution profile of BiL in TPY broth medium co‐incubated under normoxia and hypoxia conditions respectively. c,d) Digital photographs (c) and corresponding CFU statistics values (d) of the agar plates co‐incubated under normoxia and hypoxia conditions overnight. Data were presented as mean ± s.d. n = 3. Statistical significances were calculated via Student's t‐test, ***p<0.001. e,f) Digital photographs (e) and the OD_600_ values (f) of the bacterial cultures co‐incubated under normoxia and hypoxia conditions overnight. Data were presented as mean ± s.d. n = 3. Statistical significances were calculated via Student's t‐test, ***p<0.001. g) Representative digital photographs of BLB plates inoculated with the remaining bacterial cells from the major organs and tumors of mice at different time points (2, 6, 12, 24, and 72h) after injection of bacteria. h) Colony quantification of the BiL cells colonized in tumors harvested from CT26‐bearing mice at different time points (2, 6, 12, 24, and 72h) after injection of bacteria. Data were presented as mean ± s.d. n = 3. Statistical significances were calculated via Student's t‐test, ***p<0.001 and n.s. for non‐significant. i) Colony quantification of the BiL cells colonized in major organs and tumors harvested from CT26‐bearing mice at different time points (2, 6, 12, 24, and 72h) post BiL injection. Data were presented as mean ± s.d. n = 3. #: undetected.

Probiotic BiL bacterial strain has been obtained from China General Microbiological Culture Collection Center and was cultured in TPY broth medium inside the anaerobe incubator, supplied with N_2_ (90%)_,_ H_2_ (5%), and CO_2_ (5%). BiL rapidly proliferated in TPY broth under anaerobic conditions while stopped proliferating under normoxic conditions, suggesting the anaerobism (**Figure** **1**b). Similarly, BiL can be propagated on BLB agar plates with white and glossy colonies under anaerobic conditions rather than the normoxia condition (Figure 1c,d). Overnight culture of BiL yields a turbid appearance with the optical density of 0.193, corresponding to a cell density of 1.93 × 10^8^ (Figure 1e,f).

To investigate the tumor‐targeting ability of BiL, female Balb/c mice bearing subcutaneous CT26 murine colon tumor xenografts were intravenously injected with BiL at a dose of 10^6^ colony‐forming units (CFU) per mouse. Mice were then sacrificed at predetermined time points (2, 6, 12, 24, and 72 h) post‐injection with the major organs and tumor tissues dissected, homogenized, diluted (1000‐fold), and finally inoculated onto BLB plates for bacterial culture (Figure S2, Supporting Information). By counting the colonies on the plates, we found that BiL could effectively colonize and propagate inside the tumor region, reaching a bacterial density from 1 × 10^3^ CFU per gram of tissue at 2 h, to 1.15 × 10^7^ CFU per gram of tissue at 24 h. At 72 h post‐injection, the intratumoral bacterial colonization density reached 2.78 × 10^7^ CFU per gram of tissue, manifesting the effective tumor tropism (Figure 1g,h). The bacterial distributions to major organs such as the heart, liver, spleen, kidney, and lung were also evaluated. At 2 h post‐injection, BiL was dominantly distributed into the liver and spleen. Over time, the CFU values in the liver and spleen rapidly decreased and BiL was almost eliminated at 24 h post‐injection (Figure 1i). Few bacteria were accumulated inside the heart, lung, and kidney as the colonies in these organs were undetected. As contrast, no bacterial cells could be detected in the tissue homogenates dissected from mice without bacterial treatment (Figure S3, Supporting Information). In addition, ultrathin sections of tumor tissues from CT26‐bearing mice with or without BiL injections were subjected to hypoxia‐inducible factor 1a immunostaining and Gram staining respectively. Strong positive immunostaining signals of HIF‐1*α*, as well as the gram‐stains in tumor sections could be observed (Figures S4 and S5, Supporting Information), suggesting the hypoxic microenvironment inside tumor tissue and the preferential colonization of BiL inside the tumor, probably due to the presence of the hypoxic, immunosuppressive characteristics with unique biochemical features in the tumor microenvironment.^[^ ^31^ ^]^

## 3 Construction of HMME@BiL Cells with Sonodynamic Performance

**Figure 2:**
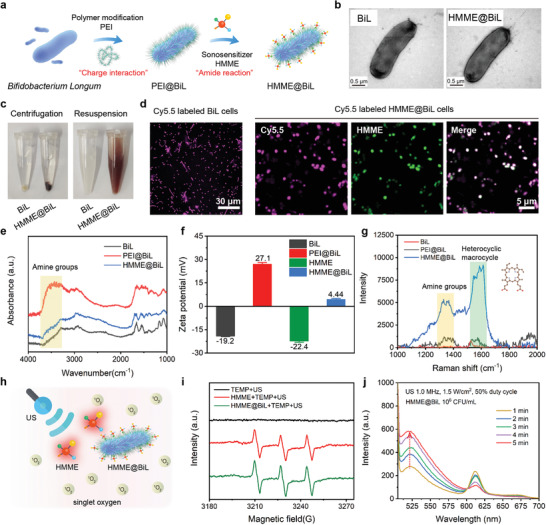
Synthesis and characterizations of the sonosensitive HMME@BiL cells. a) Schematic illustration of the construction of sonosensitive HMME@BiL cells. b) TEM image of the primitive BiL and HMME@BiL cells. c) Digital photographs of BiL and HMME@BiL cells after centrifugation and re‐suspension. d) CLSM images of Cy5.5‐labeled BiL and HMME@BiL cells. e) FT‐IR spectra of BiL, PEI@BiL, and HMME@BiL cells. f) Zeta potential profiles of BiL, PEI@BiL, HMME, and HMME@BiL cells. Data were presented as mean ± s.d., n = 3. g) Raman spectrum of BiL, PEI@BiL, and HMME@BiL cells. h) Schematic illustration of US‐triggered sonodynamic process enabled by HMME@BiL cells. i) ESR spectra of water, HMME (100 µg mL^−1^), and HMME@BiL (10^7^ CFU mL^−1^) in the presence of US irradiation (1.0 MHz, 1.5 W cm^−2^, 50% duty cycle) with TEMP as the radical trapper. j) SOSG fluorescence intensity profile of HMME@BiL under US irradiation (1.0 MHz, 1.5 W cm^−2^, 50% duty cycle) for varied durations.

The sonosensitive HMME@BiL cells were constructed based on the amide‐chemistry between BiL and clinically approved HMME sonosensitizer (**Figure** **2**a). The scanning electron microscopic image of HMME@BiL displayed a rod‐like morphology with a length of 2 µm (Figure 2b). The digital photographs of BiL and HMME@BiL showed that the color changed from turbid appearance (BiL) to dark red (HMME@BiL) after the loading of HMME (Figure 2c). We labeled the HMME@BiL by amine‐reactive Cy5.5‐NHS for confocal microscopic observation. Co‐localization of the Cy5.5 and HMME fluorescence could be observed, assembling the morphology of the BiL cells with intact rod‐shaped characters (approximately 2 µm in length and 1 µm in width) (Figure 2d). BiL cells are negatively charged due to the presence of the phosphate groups and carboxyl on the bacterial cellular outer space. Initially, we have decorated the cationic polymer PEI onto the bacterial cells based on electrostatic charge interaction. Successful decoration of the polymer was evidenced by the characteristic absorption peak at ≈3300–3500 nm assigned to amine groups in the FT‐ IR spectra (Figure 2e) and the reversed zeta potential from −19.2 mV (primitive BiL) to +27.1 mV (PEI@BiL) (Figure 2f). The emergence of the band at 1350–1400 cm^−1^ in the Raman spectra corresponded to the vibrations of ‐NH indicating that the outer surface of the formulated PEI@BiL was abundant in reactive primary amines (Figure 2g). We then conjugated the sonosensitizer HMME with carboxyl groups onto the PEI@BiL for sonosensitive HMME@BiL construction. The disappearance of the above‐mentioned amine characteristic absorption peak of BiL‐PEI after grafting with HMME in the FT‐IR spectra (Figure 2e), decreased zeta potential from +27.1 (PEI@BiL) to +4.44 mV (HMME@BiL) (Figure 2f) and the emergence of the intense character vibrations of heterocyclic macrocycle of HMME (1550–1650 cm^−1^) (Figure 2g) collectively illustrated the successful hybridization and engineering of HMME@BiL. Bacterial viability after the construction of HMME@BiL and US irradiation was also investigated. As shown in Figure S6, Supporting Information, it could be found that after the construction of the sonosensitive HMME@BiL, the bacterial viability slightly declined to 90.3%, while it continued to drop to 40.74% under US irradiation.

A typical sonodynamic process requires the ultrasound activation of the sonosensitizers to trigger the triple‐triple annihilation process between the sonosensitizer and molecular oxygen to produce the singlet oxygen species (^1^O_2_). These oxidative species are responsible to kill tumor cells with high effectiveness during in vitro and in vivo sonodynamic therapy. To monitor the ^1^O_2_ generation process, electron spin resonance (ESR) and a 1,3‐diphenylisobenzofuran (DPBF) assay were employed to evaluate the ROS generation. For ESR assays, 2,2,6,6‐tetramethylpiperidine (TEMP) was employed during all tests to serve as the radical trapper against singlet oxygen species. Typically, strong characteristic peaks of ^1^O_2_ species (amplitude of 1:1:1) could be observed in HMME group and HMME@BiL group when additional US irradiation was employed (1.0 MHz, 1.5 W cm^−2^, 50% duty cycle), while no evident peaks could be observed in the US‐only group (Figure 2i). For DPBF assay, bleaching of the chromogenic DPBF by ROS could be evaluated by recording the absorbance of the mixture at 398 nm by UV–vis spectrophotometer. As shown in Figure S8a, Supporting Information, after mixing HMME@BiL with DPBF probe, the absorption signal intensity of DPBF at 398 nm was gradually decreased with the extension of US irradiation duration (from 0 to 5 min). Next, free HMME at the same concentration was mixed with DPBF and a similar downtrend of the absorption signal intensity of DPBF could be observed (Figure S8b, Supporting Information). Singlet oxygen sensor green (SOSG), a probe that can specifically detect ^1^O_2_ was next employed to investigate the generation of ^1^O_2_ during sonodynamics (Figure 2j). In the presence of HMME@BiL, remarkable increases in SOSG fluorescence could be found under US irradiation (1.0 MHz, 1.5 W cm^−2,^ 50% duty cycle). The results of ESR, DPBF, and SOSG assay collectively indicated that the application of US could effectively stimulate the ^1^O_2_ generation enabled by sonosensitive HMME@BiL cells, facilitating the cellular SDT and animal SIT modalities.

## 4 Cellular SDT Performance and Immune Responses Enabled by HMME@BiL Cells

**Figure 3:**
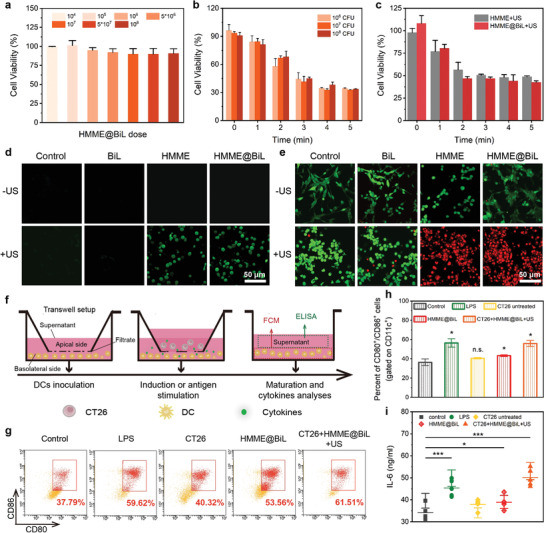
In vitro cytotoxicity of HMME@BiL cells against CT26 cells and cellular immune response stimulation. a) Relative cell viabilities of MRC‐5 cells co‐incubated with HMME@BiL. Data were presented as mean ± s.d., n = 6. b) Relative cell viability of CT26 cells incubated with HMME@BiL (10^6^, 10^7^, 10^8^ CFU) under US irradiation of varied durations. Data were presented as mean ± s.d., n = 6. c) Relative cell viability of CT26 cells incubated with HMME@BiL (10^6^ CFU) and free HMME at the same HMME concentration under US irradiation of varied durations. Data were presented as mean ± s.d., n = 6. d) CLSM images of CT26 cells stained with DCFH‐DA after different treatments: control, BiL only, HMME only, HMME@BiL only, US only, BiL + US, HMME + US and HMME@BiL + US; e) CLSM images of CT26 cells stained with Calcein‐AM and PI after various treatments: control (without any treatment), BiL only, HMME only, HMME@BiL only, US only, BiL + US, HMME + US and HMME@BiL + US; f) Schematic illustration of the in vitro transwell setup and co‐incubation details: CT26 cells and their residues were placed in the apical chamber and DCs were cultured in the basolateral chamber; g) Flow cytometric results of the DCs stained with anti‐CD11c FITC, anti‐CD80 APC and anti‐CD86 PE for maturation identification. h,i) Quantification of the level of DC maturation (h) and the secretion of IL‐6 (i) in DC suspensions. Data are expressed as mean ± s.d. (n = 4). Statistical significances were calculated via Student's t‐test, *p<0.05, **p<0.01, ***p<0.001 and n.s. for non‐significant.

After demonstrating the sonodynamic performance of the constructed sonosensitive HMME@BiL cells under US irradiation, we next explore the in vitro SDT performance of HMME@BiL at the cellular level. The cytocompatibility of HMME@BiL was initially evaluated in human embryonic lung fibroblasts MRC‐5 cells by Alamar blue cell viability assay kit (**Figure** **3**a). The results indicate that no significant cytotoxicity for HMME@BiL at varied concentrations from 10^4^ CFU to 10^8^ CFU. We then investigated the therapeutic outcome of the HMME@BiL on murine colon cancer CT26 cell line with 10^6^, 10^7^, and 10^8^ CFU under US irradiation. The cytotoxicity profiles were majorly dependent on the irradiation durations rather than the doses of HMME@BiL cells (Figure 3b). Following the biosafety consideration, we then employ 10^6^ CFU as the optimal dose during in vitro cellular investigation. With the extension of US irradiation duration, the cell viability decreased from 93% to 57% and from 61% to 40% respectively at the US intensity of 1.0 and 1.5 W cm^−2^ (Figure S11, Supporting Information). HMME at identical HMME concentration was also evaluated to compare the in vitro cancer cell ablation efficiency between free HMME and HMME@BiL (Figure 3c). No significant differences could be found between two groups. These results showed that the cell‐killing performance is typically dependent on irradiation time and intensities. In the present experimental design, HMME@BiL at the dose of 10^6^ CFU, US intensity of 1.5 W cm^−2^, and irradiation duration of 5 min could effectively cause tumor cell death.

To investigate the underlying mechanism of in vitro SDT enabled by sonosensitive HMME@BiL cells, the intracellular ROS production level was indicated by a specific ROS marker 2′‐7′‐dichlorofluorescein diacetate (DCFH‐DA). Specifically, CT26 cells were co‐incubated with PBS, BiL (10^6^ CFU), HMME (100 µg mL^−1^), and HMME@BiL cells (10^6^ CFU) respectively for 4 h with subsequent exposure to the US (1.0 MHz, 1.5 W cm^−2^, 50% duty cycle) for 5 min. DCFH‐DA stained cells were subjected to confocal microscopic observations. From the images, both HMME and HMME@BiL treated groups could produce a large amount of ROS after US irradiation, as revealed by the strong intracellular green fluorescence inside the cells (Figure 3d). Comparatively, undetected ROS production could be observed for BiL‐treated CT26 cells, as revealed by the low fluorescence intensity profile. Next, viable/dead dual‐staining assay was used for cell‐staining to evaluate the cell‐killing effect in various treatment groups: PBS, BiL (10^6^ CFU), HMME (100 µg mL^−1^), and HMME@BiL cells (10^6^ CFU). Green fluorescence could be observed in all the groups with different treatments when no US irradiation was applied. In PBS and BiL groups, few CT26 cells were stained with red fluorescence after US irradiation, implicating the biocompatibility of the US treatment modality without sonosensitizer. On the contrary, in the presence of the sonosensitizer, HMME and HMME@BiL groups showed bright red fluorescence after US irradiation, indicating that the tumor cells were destructed under SDT (Figure 3e). Next, we employed a FITC‐labeled TUNEL apoptosis assay kit to detect the apoptotic cell death during therapeutics. As shown in Figure S12, Supporting Information, both HMME and HMME@BiL treated groups could induce the apoptosis percentages up to 59.6% and 61.1% against CT26 cells respectively. From the above results, it could be inferred that SDT enabled by HMME@BiL cells could offer prominent tumor‐killing ability with high biocompatibility.

It has been reported that sonodynamic therapy could effectively trigger ICD against the tumor cells by presenting the tumor cell fragments to the immune cells.^[^ ^23^, ^32^ ^]^ Dendritic cells are responsible for the antigen‐presenting characters to activate the naive T cells, playing a crucial role in both innate and adaptive immune responses within the host.^[^ ^33^ ^]^ When exposed to the antigens, the immature DCs would engulf antigens and present the antigens into peptides as they migrate to the nearby lymph nodes. Subsequently, the immature DCs go through maturation and present the complex peptide to the naive T cell, stimulating the T cells for differentiation.^[^ ^34^ ^]^ To investigate the SDT‐enabled activation of immature DCs, bone‐marrow‐derived DCs were evaluated by detecting the upregulations of co‐stimulatory molecules CD80/CD86 (representative DC maturation markers) by flow cytometry. DCs were seeded in the basolateral chamber and co‐incubated with 1) PBS, 2) LPS (lipopolysaccharide, 1 µg mL^−1^), 3) CT26 (10^7^), 4) HMME@BiL cells (10^6^ CFU), 5) CT26 (10^7^) + HMME@BiL cells (10^6^ CFU) + US respectively in the apical chamber overnight (Figure 3f). From the cytometric results, the population of CD80^+^CD86^+^ subset (55.96%) in the cells treated with CT26 (10^7^) + HMME@BiL cells (10^6^ CFU) + US was significantly higher than that of the cells in the untreated control group (36.42%). The population was comparable to that of the DCs stimulated by LPS (positive control) (56.48%) (Figure 3g,h). The CD80^+^CD86^+^ populations in CT26 group (40.65%) and HMME@BiL group (43.36%) had no significant differences as compared to the untreated group. Cytokine secretion by DCs is another character dictating the maturation process of DCs.^[^ ^35^ ^]^ By measuring the cytokine IL‐6 content in the supernatant of DCs by Enzyme‐Linked ImmunoSorbent Assay, a remarkable increase could be indicated in the sample subjected to CT26 (10^7^) + HMME@BiL cells (10^6^ CFU) + US treatment group (1.3 fold increase) and LPS treatment group (1.2 fold increase) respectively as compared to the control group (Figure 3i). Therefore, it could be assumed that the SDT enabled by HMME@BiL cells could trigger the release of the tumor antigen to an extent to promote DC maturity and further enhancement of the antitumor response of T cells. Nevertheless, such immune responses demand further improvements for better therapeutic consequences by immune‐agonists.

Encouraged by the in vitro cellular antitumor performance, in vivo experiments were scheduled according to the guidelines of the Laboratory Animal Ethics Committee of Shanghai Tenth People's Hospital (SHDSYY‐2021‐6600). We first conducted the biosafety assessment. The selective distribution of BiL inside the tumor tissues has implied the biocompatibility and biosafety of the bacteria. For a comprehensive investigation, healthy female ICR mice (5 weeks) were divided into four groups and treated with 1) PBS; 2) BiL (i.v., 10^6^ CFU); 3) HMME@BiL (i.v., 10^6^ CFU), and 4) HMME@BiL (i.v., 10^8^ CFU), respectively. From the bodyweight curves during the evaluation, it could be found that a slight weight loss could be observed at 48 h post‐injection for BiL and HMME@BiL injection groups (Figure S13a, Supporting Information). The bodyweight of mice eventually recovered to healthy status as compared to the control group over time. Transient bacteremia might result in a change in body weight in the bacterial injection groups. At day 15 post‐injection, mice were sacrificed with their blood collected for hematological and biomedical index analysis. Most of the indexes were non‐significantly distributed as compared to the untreated healthy mice, demonstrating the high hematological biocompatibility (Figure S13b–i, Supporting Information). Higher plasma platelet counts could be observed for mice treated with HMME@BiL at the doze of 10^8^ CFU than in the control group (Figure S13d, Supporting Information). The possible reason for this regulation might be attributed to the immune response against the bacterial cells when a high dose of bacteria was employed to activate the aggregation of platelets.^[^ ^36^ ^]^ Ultrathin sections of main organs including heart, liver, spleen, lung, and kidney from mice with different treatments were subjected to hematoxylin‐eosin (H&E) staining (Figure S14, Supporting Information). No noticeable damage to the main organs was detected in all the different treatment groups, indicating the injection of BiL at the dose below 10^6^ CFU was biocompatible and tolerable for mice.

## 5 In Vivo Animal SIT Enabled by HMME@BiL with STING Agonist

**Figure 4:**
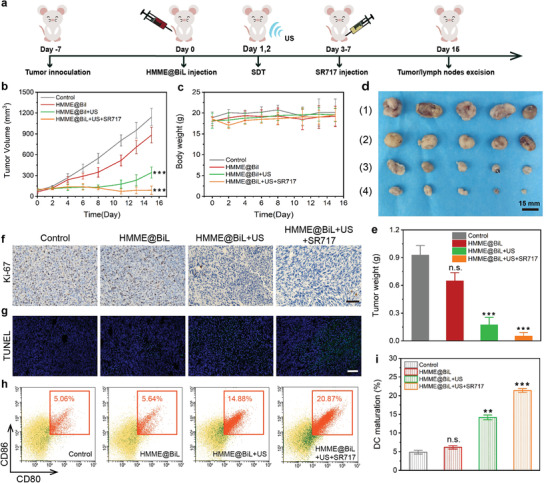
In vivo animal anti‐tumor investigation by HMME@BiL cells‐enabled SDT and immunotherapy. a) Schematic illustration of the in vivo treatment schedule during the anti‐tumor experiment. b) Tumor growth curves of mice from different groups after various treatments as indicated. Data were presented as mean ± s.d. n = 5. Statistical significances were calculated via Student's t‐test, ***p<0.001. c) The body weight curves of mice after different treatments during the in vivo anti‐tumor investigation period. Data were presented as mean ± s.d. n = 5. d) Digital photographs of excised tumors of mice from each group at the end of the anti‐tumor investigation period: 1) Control, 2) HMME@BiL, 3) HMME@BiL+US, and 4) HMME@BiL+US+SR717. e) Mean mass profile of the tumors dissected from mice from each group at the end of the anti‐tumor investigation period. Data were presented as mean ± s.d. n = 5. Statistical significances were calculated via Student's t‐test, ***p<0.001 and n.s. for non‐significant. f) Microscopic images of antigen Ki‐67‐stained tumor ultrathin sections of mice from each group. g) Fluorescent microscopic images of TUNEL‐stained tumor ultrathin sections of mice from each group. h,i) Flow cytometric results (h) and corresponding statistics (i) on DC maturation in the tumor‐draining lymph nodes dissected from mice from each groups. Cells were stained with anti‐CD11c FITC, anti‐CD80 APC, and anti‐CD86 PE. Data were presented as means ± s.d. n = 3. Statistical significances were calculated via Student's t‐test, **p<0.01, ***p<0.001 and n.s. for non‐significant.

We next explored the in vivo therapeutic effect enabled by HMME@BiL under US irradiation. Based on the stimulated ICD by the SDT, we speculated that the combination of SDT and immunotherapy could play a synergistic effect on tumor therapy. SR717 as a recently‐developed STING agonist was adopted in this work, which displays the antitumor effect by promoting the activation of CD8^+^ T natural killer cells and dendritic cells in relevant tissues. CT26 cells (1 × 10^6^) were subcutaneously injected into the right abdomen of each mouse to investigate the in vivo therapeutic effects on Balb/c mice (**Figure** **4**a). When the tumor volume reached around 80 mm^3^, the mice were randomly divided into four groups (n = 5): 1) PBS; 2) HMME@BiL; 3) HMME@BiL+US; and 4) HMME@BiL+US+SR717. For mice from each group, BiL and HMME@BiL cells were intravenously injected at the dose of 10^6^ CFU on day 0. US irradiation was conducted twice at 24 and 48 h post‐i.v. injection for mice in the HMME@BiL + US group and HMME@BiL + US + SR717 group under anesthesia. After the second irradiation, mice in HMME@BiL + US + SR717 group were intraperitoneally injected with STING agonist SR717 at a daily dose of 30 mg kg^−1^ for five consecutive days. From the xenograft growth curve, we could find that a similar therapeutic effect could be observed for mice receiving HMME@BiL + US and HMME@BiL + US + SR717 treatments at the initial 8 days (Figure 4b). The suppression effect for mice receiving HMME@BiL + US treatment was gradually decreased, as revealed by the rebounded tumor growth. The administration of SR717 brought a stronger and more durable tumor suppression effect as compared to the HMME@BiL + US group. There was no significant difference between the experimental groups and control groups, as revealed from the bodyweight profile (Figure 4c). A slight body temperature increase of about 0.2 °C could be observed for mice from HMME@BiL cells injected groups at 2 days post‐injection (Figure S16, Supporting Information), which could be attributed to the transient bacteremia. No significant differences could be observed in HMME@BiL + US group and HMME@BiL + US + SR717 group, indicating that the application of SR717 had no obvious side effects against the host immune system. On day 15, mice were sacrificed and the tumor xenografts and lymph nodes were resected for detailed analysis. An interesting phenomenon could be found that the tumor xenografts of mice from HMME@BiL group had a smaller volume and weight than the PBS group albeit no US irradiation was applied. A possible reason might be that the BiL cell colonization in the tumor could constrain the growth of the tumor xenograft. The average tumor volume and tumor weight of HMME@BiL + US group and HMME@BiL + US + SR717 group were significantly smaller than that of the control group (Figure 4e, Figure S17, Supporting Information), revealing the excellent tumor‐targeted ability and SDT therapeutic effect in tumor suppression based on sonosensitive HMME@BiL. In addition, the volume and weight of tumor xenografts in HMME@BiL + US + SR717 group were much smaller than those in HMME@BiL + US group (tumor volume: 343 mm^3^ ± 80.9 mm^3^ for group 3, 88.8 mm^3^ ± 60.0 mm^3^ for group 4). It could be shown that although sonodynamic therapy enabled by HMME@BiL could effectively suppress the growth of the tumor, the SDT‐triggered ICD was unable to contribute to potent immune responses of the hosts. While the addition of STING agonist SR717 could activate the anti‐tumor immunity for prominent tumor suppression and eradication. Ultrathin sections of tumor tissues were subjected to TUNEL assay and ki‐67 detection kit respectively. A major proportion of ki‐67 positive cells (brown) could be observed in the control group and HMME@BiL group while almost no ki‐67 positive cells could be found in groups 3 and 4 (Figure 4f). In addition, we could observe bright green fluorescence (TUNEL positive cells) in HMME@BiL + US and HMME@BiL + US + SR717 group as compared to the PBS group (Figure 4g), collectively indicating that HMME‐BiL enabled SDT could effectively inhibit the proliferation of the malignant tumor cells, meanwhile inducing cell apoptosis. To further investigate the in vivo immune responses, DCs were isolated from tumor‐draining lymph nodes and their maturation markers (CD80/CD86) were assessed by flow cytometry (Figure 4h,i). These results showed that the proportion of CD80/86 positive cells in HMME@BiL + US group increased from 4.85% of the control group to 14.18%. While in HMME@BiL + US + SR717 group it increased to 21.38%, much higher than that of the HMME@BiL + US group. These results indicated that DCs could be activated by sonodynamic therapy in vivo, while combined tumor immunotherapy could enhance the activation level due to the synergy of SDT and immune agonist SR717, contributing to the prominent in vivo SIT modality.

**Figure 5:**
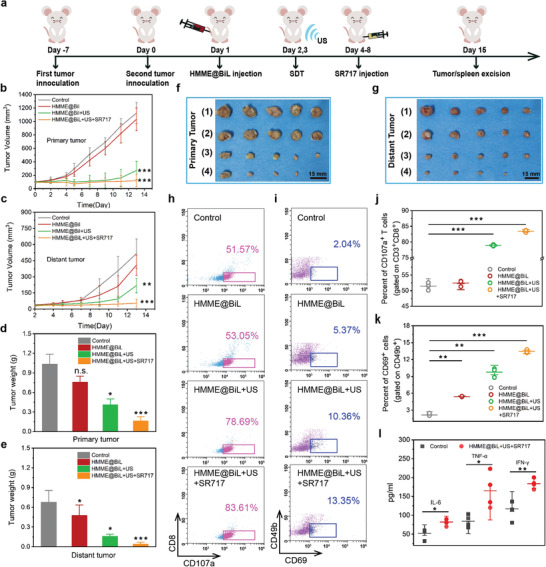
Synergistic HMME@BiL cells‐augmented SDT and immunotherapy for in vivo anti‐tumor investigation in a bilateral subcutaneous xenograft model. a) Schematic illustration of the schedules for the in vivo anti‐tumor investigation. Mice bearing CT26 subcutaneous tumors on both sides were used in this experiment. Tumors on the right side of the abdomen were designed as “primary tumor” for SDT, and those on the left side were set as “mimic distant tumors” without SDT**;** b,c) Primary tumor growth curves (b) and distant tumor growth curves (c) of mice from different groups as indicated in the figure. Data were presented as mean ± s.d. n = 5. Statistical significances were calculated via Student's t‐test, **p<0.01 and ***p<0.001 d,e) Mean mass profile of primary (d) and distant tumors (e) dissected from mice from different treatment groups at the end of anti‐tumor investigation period. Data were presented as mean ± s.d. n = 5. Statistical significances were calculated via Student's t‐test, *p<0.05, ***p<0.001 and n.s. for non‐significant. f,g) Digital photographs of excised primary (f) and distant (g) tumors of mice from different treatment groups at the end of anti‐tumor investigation period. h–k) Flow cytometric results of CD107a expression gated on CD8^+^ T cells (h) and CD69 expression gated on CD49b^+^ NK cells (i) isolated from spleens of CT26 bearing mice and the corresponding statistical results (j,k). Data were presented as mean ± s.d. n = 3. Statistical significances were calculated via Student's t‐test, **p<0.01, ***p<0.001 and n.s. for non‐significant. l) ELISA assays on cytokine levels of IL‐6, TNF‐*α*, and IFN‐*γ* in serum isolated from mice in control group and HMME@BiL + US + SR717 group. Data were presented as mean ± s.d. n = 4 Statistical significances were calculated via Student's t‐test, *p<0.05 and**p<0.01.

Next, a bilateral subcutaneously tumor model mimicking human metastatic cancer was employed to evaluate the distant antitumor immunity. According to the experiment design and schedule (**Figure** **5**a), the primary tumor xenograft was inoculated into the right abdomen as the primary tumor. When the primary tumor volume reached around 100 mm^3^, a second tumor xenograft was inoculated into the left abdomen as an artificial distant metastatic tumor. Mice were randomly divided into four groups (n = 5): 1) Control, 2) HMME@BiL, 3) HMME@BiL + US, 4) HMME@BiL + US + SR717. BiL and HMME@BiL cells were intravenously injected at the dose of 10^6^ CFU on day 1. US irradiation was conducted twice at 24 and 48 h post‐i.v. injection for mice in HMME@BiL + US group and HMME@BiL + US + SR717 group under anesthesia. After the second irradiation, mice in HMME@BiL + US + SR717 group were intraperitoneally injected with STING agonist SR717 at a daily dose of 30 mg kg^−1^ for five consecutive days. Treatment results of different groups against the primary and mimetic distant tumors were obtained (Figure 5b–g and Figure S18a, b, Supporting Information). Administration of HMME@BiL alone did not exhibit a therapeutic effect on the inhibition of distant tumors. Although HMME@BiL‐augmented SDT could suppress the primary tumor growth, it failed to exert influence on the distant tumor. Notably, HMME@BiL‐augmented SDT combined with SR717 not only destruct the major lesion of the primary CT26 tumor (Volume Suppression Rate of 92%) but also significantly suppressed the distant tumor growth (Volume Suppression Rate of 72%). The variation trend of tumor volume was consistent with that of the dissection weight of the tumor, as indicated in Figure 5b‐e, wherein HMME@BiL‐augmented SDT combined with SR717 yielded the largest reduction of tumor weight, in both primary (84%) and distant (76%) tumors.

To understand the underlying mechanism of the antitumor effects triggered by SIT, immune cells in the spleens were assessed on the 13th day after the first treatment using a bilaterally subcutaneous tumor model. Flow cytometry was used to measure the activation level of spleen lymphocytes. The quantity of cytotoxic T cells labeled by CD3^+^CD8^+^ was statistically increased in the group treated by HMME@BiL + US + SR717, as shown in Figure S19, Supporting Information. CD107a, also being recognized as lysosome‐associated membrane protein 1 (LAMP‐1), encodes a membrane glycoprotein that provides selectins with carbohydrate ligands and can be used to evaluate the cytotoxic function of CD8^+^ T cells. The FCM results showed that the proportion of CD107a^+^ T cells in the CD8^+^ T cells were 83.45% and 51.42% in HMME@BiL + US + SR717 group and control group respectively (Figure 5h,j). A significant increase in the proportion of CD107a in HMME@BiL + US + SR717 group revealed that cytotoxic T cells degranulation was effectively triggered under this treatment. CD69, also being recognized as an activation inducer molecule or early activation antigen, is the earliest inducible cell surface glycoprotein acquired during lymphoid activation involved in early events of NK cells, was also quantified by flow cytometry. The proportion of CD69^+^ cells in CD49b^+^ NK cells was improved from 2.15% to 13.43% in HMME@BiL + US + SR717 group (Figure 5i–k). In addition, the quantity of NK cells in SDT plus SR717 group increased from 1.91% to 4.79% compared to the control group (Figure S20, Supporting Information). The results of flow cytometry of NK cells illustrated that SIT treatment both raised the quantity and the degree of activation of NK cells. Next, we attempted to further identify the possible mechanism of the antitumor immunity post combined tumor immunotherapy by analyzing the related cytokines. On day 13, mice were sacrificed and serum was extracted to perform an ELISA test on IL‐6, TNF‐*α*, and *γ*‐IFN, which play vital roles in cellular immunity against cancer. It could be observed that as compared with the control group, a significant enhancement was achieved in the concentration of cytokines in HMME@BiL + US + SR717 group. The concentration increased from 52.09 to 82.13 pg mL^−1^ in IL‐6, from 84.27 to 164.64 pg mL^−1^ in TNF‐*α*, and from 117 to 183.82 pg mL^−1^ in IFN‐*γ* respectively indicating the establishment of strong antitumor immune responses (Figure 5l).

## 6 Conclusion

In conclusion, we reported on the rational construction of clinically approved HMME engineered tumor‐targeting probiotic BiL cells for highly efficient SIT with biocompatibility. Prominent sonodynamic performance has been investigated for HMME@BiL cells at both in vitro cellular and in vivo animal levels. Based on the satisfied tumor tropism of the BiL, the engineering HMME@BiL cells could facilitate the effective SDT therapeutics upon US irradiation. We also demonstrated that HMME@BiL‐augmented SDT in combination with SR717 not only efficiently suppressed the primary tumor growth but also substantially prevented the mimetic distant metastasis. Systematic immune responses, including elevation of enhanced DC maturation and cytokine secretion, as well as the upregulation of the activation of CD8^+^ T cells and NK cells, have been shown to contribute to the enhanced comprehensive SIT against malignant tumors. The present work demonstrated the synergy of probiotic microbe‐based tumor‐targeted sonodynamic therapy and active immunotherapy against malignant tumors with metastatic features, illuminating the promising tumor therapeutics based on the conceptual advances of microbiotic nanomedicine.

Statistical Analysis: Data significance is calculated according to the Student's t‐test: *P < 0.05, **P < 0.01, and ***P < 0.001. n.s. for nonsignificant. Data presentation and sample size are indicated in the figure legend.

## Conflict of Interest

The authors declare no conflict of interest.

## Supporting information

Supporting Information

## Data Availability

The data that support the findings of this study are available in the supplementary material of this article.
