# Supplementary material for: Probiotic Engineering and Targeted Sonoimmuno‐Therapy Augmented by STING Agonist
Source: Adv Sci (Weinh). 2022 May 23;9(22):2201711. doi: 10.1002/advs.202201711 (PMC9353485; doi:10.1002/advs.202201711)
Supplement: Supplementary file 1 — Supporting Information [file ADVS-9-2201711-s001.pdf]

ADVANCED SCIENCE
Open Access

Supporting Information

for *Adv. Sci.*, DOI 10.1002/advs.202201711

Probiotic Engineering and Targeted Sonoimmuno-Therapy Augmented by STING Agonist

Dan Lu, Liying Wang, Liping Wang, Liwei An, Minfeng Huo*, Huixiong Xu* and Jianlin Shi*

Supporting Information

Probiotic Engineering and Targeted Sonoimmuno-Therapy Augmented by STING Agonist

Dan Lu, Liying Wang, Liping Wang, Liwei An, Minfeng Huo*, Huixiong Xu* and Jianlin Shi*

Contents:

1. Experimental Section

2. Supplementary Figures

1. Experimental Section

Materials. All the solvents were purchased from Aladdin Co., Ltd. (China). The TPY broth medium and the BLB media were purchased from Hopebio Co., Ltd. (China). Polyetherimide and EDC were purchased from Aladdin Co., Ltd. (China). DCFH-DA and TEMP were purchased from Sigma Co., Ltd. (USA). HMME was purchased from Shanghai Yuanye Technology Co., Ltd.. SOSG was purchased from Thermo Fisher Scientific (USA). Alamar Blue was purchased from Yeason Co., Ltd. (China). DCFH-DA and Calcein AM/PI were purchased from Dojindo Co., Ltd. (Japan). Lipopolysaccharid, GM-CSF and IL-4 were purchased from MedChemExpress Co., Ltd. (China). SR717 was purchased from Selleck Co., Ltd. (China). All the antibodies were purchased from Abcam Co., Ltd. (USA).

Bacteria culture. *Bifidobacterium Longum* was obtained from China General Microbiological Culture Collection Center and stored at −80 °C. For bacterial culture, BiL was streaked on the bacteria BLB solid plate and then incubated at 37 °C for overnight. Bacteria colonies were picked out and grown overnight in TPY broth medium in an anaerobic incubator at 37 °C. Afterward, the bacteria-containing medium was diluted by 50-folds into fresh medium and further grown to the early stationary phase. Thereafter, the bacteria were collected by centrifugation (4000 rpm, 10 min) and diluted with sterile PBS for further experiments.

Fabrication of sonosensitive HMME@BiL cells. Sonosensitive HMME@BiL cells were synthesized based on the Amide chemistry. In brief, bacteria colonies were allowed to grow overnight in TPY broth medium in an anaerobic incubator at 37 °C. Afterward, the bacteria were collected by centrifugation (4000 rpm, 10 min) and redispersed in 1 mL PBS (pH = 7.4). 1 mL solution containing Polyetherimide (0.05 g/mL) was added into the above bacterium solution (10^9 CFU) slowly and stirred for 2 h. PEI@BiL was then obtained by centrifugation (4000 rpm, 10 min) and suspended in 10 mL MES buffer (pH = 6.0). 150 mg EDC dispersed in 1.7 ml MES buffer (pH = 6.0) and 3 mg HMME dispersed in DMSO were mixed and stirred for 40 min and then added into 1 mL PEI@BiL solution for the further reaction for 1 h. The product was centrifuged and washed with PBS three times to remove the residual free HMME.

***In vivo* animal experiments.** Female BALB/c mice (5 weeks) were purchased from Shanghai SLAC Laboratory Animal Co. Ltd. and the experiment was performed according to the guidelines of the Laboratory Animal Ethics Committee of Shanghai Tenth People's Hospital (SHDSYY-2021-6600).

***In vivo* tumor xenograft establishment.** To establish the murine tumor xenograft models, CT26 cells (1×10^6) were subcutaneously implanted into BALB/c mice. After 7 days, the volume of the xenografts grew to ≈ 100 mm^3.

***In vivo* bacterial colonization.** Female Balb/c mice bearing subcutaneous CT26 murine colon tumor xenografts were intravenously injected with BiL at dose of 10^6 CFU per mouse. Mice were then sacrificed at predetermined time points (2, 6, 12, 24, and 72 h) The major organs of mice with bacterial injection were collected at predetermined time points (2, 6, 12, 24, and 72 h). Major organs, including heart, liver, spleen, lung, kidney, and tumor, were then weighted and homogenized at 4°C in sterile PBS (pH = 7.4). Those samples were diluted (1000-fold) and inoculated on LB plates. After 12 hours of incubation, bacterial colonies were counted. The bacterial accumulation profiles (CFU per gram of tissue) were calculated with colony counts per gram of tissue weight.

ESR assays. To evaluate the generation of $^1O_2$, TEMP was employed as a trapper during the ESR assays for singlet oxygen species identification. HMME@BiL cells (10^7 CFU) were suspended in 1 mL PBS (pH = 7.4), followed by the addition of TEMP (10 μL, 97 μM). US irradiation (1.0 MHz, 1.5 W/cm^2, 50 % duty cycle) was employed to the mixture for 5 mins.

The $^1O_2$ generation was detected by ESR spectrometer. HMME@BiL + TEMP and US + TEMP were also tested for comparison.

DPBF assays for singlet oxygen species quantification. HMME@BiL cells (10^7) were suspended in PBS (pH = 7.4), followed by the addition of DPBF (40 μL, 8 mM). Then, the mixture was exposed to US irradiation (1.0 MHz, 1.5 W/cm^2, 50 % duty cycle) for different periods in dark. The absorption intensity of DPBF at 398 nm was measured by a UV-Vis spectroscope.

***In vitro* cellular antitumor activity.** CT26 cells were seeded in 96-well plates (1 × 10^5 cells per well) and cultured for 12h. Cells were then rinsed with PBS and replaced with 100 μL RPMI 1640 medium containing HMME@BiL (10^6 CFU) per well with subsequent US irradiation (1.0 MHz, 1.5 W/cm^2, 50 % duty cycle) for different periods. After 4 h, the relative cell viabilities were detected by an Alamar Blue assay following the protocol of the kit. For CLSM live/dead observations, CT26 cells (1 × 10^5 cells) were plated on a CLSM-exclusive culture disk (φ = 15 mm, Corning Inc., NY, USA), and cultured for 12 h to facilitate adherence of cells. Then, CT26 cells were treated for 24 h by the following counterparts: Control, US, BiL (10^6 CFU), BiL+US (10^6 CFU), HMME, HMME+US, HMME@BiL (10^6 CFU), HMME@BiL+US (10^6 CFU). Subsequently, these cells were stained with Calcein-AM/PI followed by observation using CLSM.

***In vitro* ^{1}O$_2$ generation at the cellular level.** The intracellular $^1O_2$ level was measured by the ROS-sensitive fluorescent probe DCFH-DA, which can emit bright green fluorescence after being oxidized by $^1O_2$ species upon intercellular internalization. Cells were subjected to indicated treatments (i.e., control, HMME, HMME@BiL, US, HMME+US and HMME@BiL + US) for 4 h after cell attachments. Then, the culture medium was replaced by DCFH-DA (100 μL, 10 % in RPMI 1640 medium) and subjected to US irradiation (1.0 MHz, 1.5 W/cm^2, 50% duty cycle, 5 min). After incubating for another 30 mins, the cells were washed with PBS two times and then subjected to CLSM observation.

***In vitro* DC stimulation in transwell experiment.** Bone-marrow-derived DCs were isolated from 6-weeks-old BALB/c mice according to an established method. DCs were seeded in the basolateral chamber and co-incubated with CT26 (10^7), HMME@BiL cells (10^6 CFU), CT26 (10^7) + HMME@BiL cells (10^6 CFU) + US respectively in the apical chamber.

Lipopolysaccharide (LPS, obtained from MedChemExpress) at a dose of 1 μg/mL was used as the positive control. And PBS (pH = 7.4) was used as a negative control. After various treatments, DCs stained with anti-CD11c FITC, anti-CD80 APC and anti-CD86 PE were analyzed by flow cytometry (Beckman Coulter). The proinflammatory cytokines IL-6 from DC suspension were tested by ELISA kits with a standard protocol.

***In vivo* biocompatibility assays.** Four randomly divided groups (five mice per group) of healthy ICR mice were treated with PBS, BiL cells (5×10^6 CFU per mouse) and HMME@BiL cells (5×10^6 CFU and 5×10^8 CFU per mouse) respectively. The body weights of mice were recorded every two days. After the 15-days biocompatibility evaluation period, mice were sacrificed and the blood was sampled for hematological and biomedical index analysis. Major organs (heart, liver, spleen, lung, and kidney) of mice from different groups were harvested for hematoxylin and eosin (H&E) staining for pathological evaluation.

***In vivo* anti-tumor sonodynamic therapy.** Twenty tumor-bearing mice were randomly divided into 4 groups (n =5) including the following: (1) saline, (2) HMME@BiL, (3) HMME@BiL + US, (4) HMME@BiL + US + SR717. When tumor volumes grew to around 80 mm^3, mice were intravenously injected via tail veins accordingly, with an identical BiL dose of 10^6 CFU on day 0. US irradiations in the above groups were performed at 24 and 48 h post-injection. Body weights and tumor volumes of mice were monitored every two days. At the end of the *in vivo* anti-tumor evaluation for 15 days, mice were sacrificed with their tumors excised, weighed, and photographed. In addition, tumors were collected for H&E, TUNEL and antigen Ki-67 staining. Tumor-draining lymph nodes were excised for flow cytometric analyses after co-staining with anti-CD11c FITC, anti-CD80 APC and anti-CD86 PE.

***In vivo* combination anti-tumor therapy in a bilateral model.** Bilateral tumor xenograft murine models were established by subcutaneously injecting the CT26 cells (1×10^6) into both side abdomen of each mouse at day 0 and day 7 respectively. Mice were randomly divided into four groups (n = 5): (1) saline, (2) HMME@BiL, (3) HMME@BiL + US and (4) HMME@BiL + US + SR717. Accordingly, HMME@BiL cells were intravenously injected into the mice at the dose of 1×10^6 CFU on day 0. US irradiations were performed at 24 and 48 h post-injection. SR717 at the dose of 30 mg/kg was administered on days 2-5. The tumor volume was calculated according to the following formula: ($width^2 \times length$) / 2, as measured

by the digital caliper. At the end of the experiment, mice were sacrificed with their tumors excised, weighed and photographed. Alternatively, spleens of mice from different groups were dissected at 48 h post-injection and treated with the spleen lymphocyte dissociation kit (Solarbio) to produce a single-cell suspension according to the procedures. The harvested cells were further stained with several fluorochrome-conjugated antibodies including CD3-violetFluor^{TM}450 (TONBO, Catalog: 75-0032-U100), CD8a-APC-Cyanine7 (TONBO, Catalog: 25-0081-U100), CD49b-FITC (Biolegend, Catalog:103503), CD69-PE-Cyanine7 (TONBO, Catalog: 60-0691-U100), CD107a-PE (Thermo/eBio, Catalog: 12-1071-82), followed by flow cytometric analyses. All antibodies were diluted for 100 times. Blood samples were collected and the proinflammatory cytokines, including γ-IFN, IL-6 and TNF-α serum, were tested by ELISA.

2. Supplementary Figures

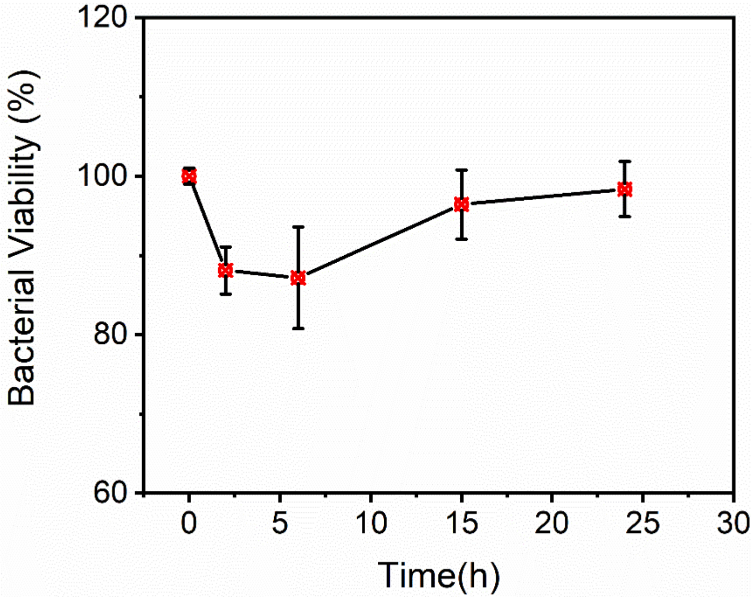

Figure S1. The bacterial viability of BiL cells under aerobic conditions. Data were presented as mean ± s.d. n = 4.

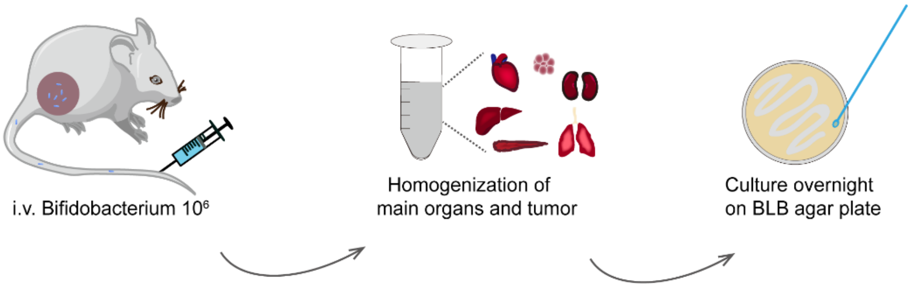

Figure S2. Schematic illustration of the process of *in vivo* bacterial colonization.

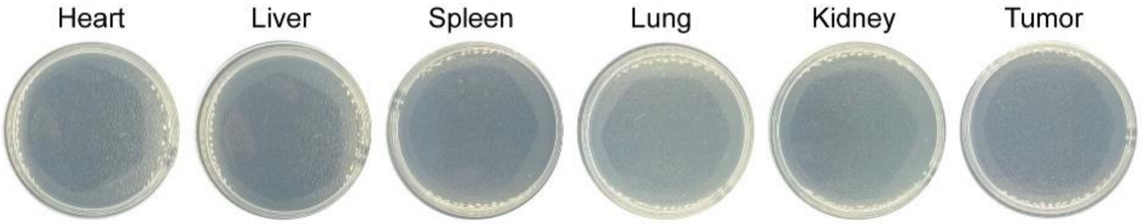

Figure S3. Representative digital photographs of BLB plates inoculated with tissue homogenate diluent of the major organs and tumor of CT26-bearing mice without treatment.

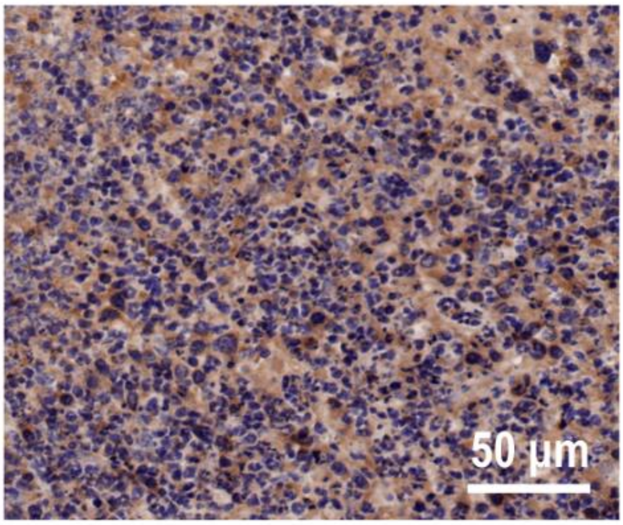

Figure S4. Microscopic images for HIF-1α staining tumor section.

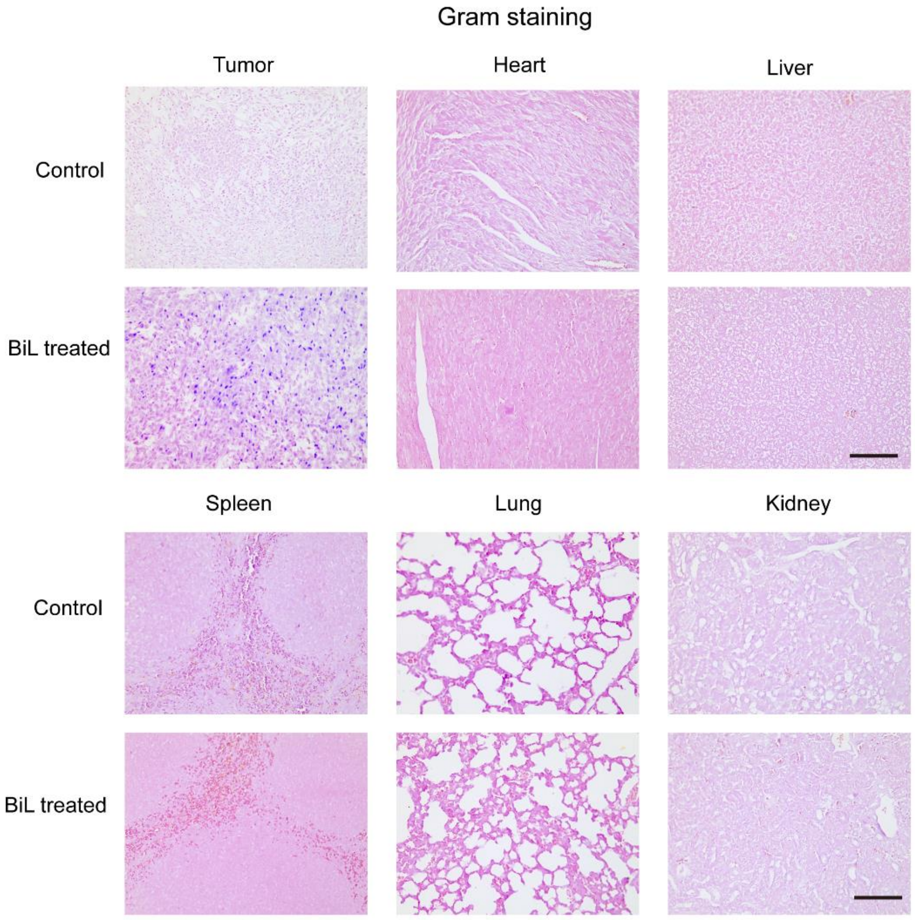

Figure S5. Microscopic images for Gram staining of tumor and main organs sections with or without *in vivo* BiL administrations (scale bar = 100 μm).

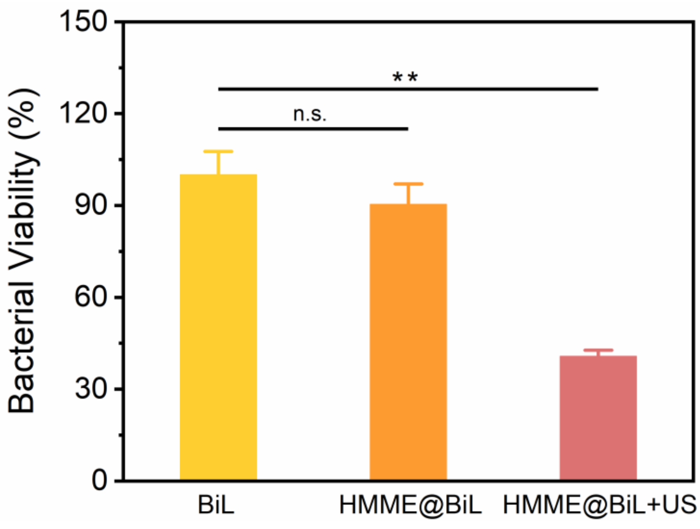

Figure S6. Bacterial viability after the construction of HMME@BiL and US irradaition. Data were presented as mean ± s.d. n = 4. Statistical significances were calculated via Student's t test, $^{**}p<0.01$ and n.s. for non-significant.

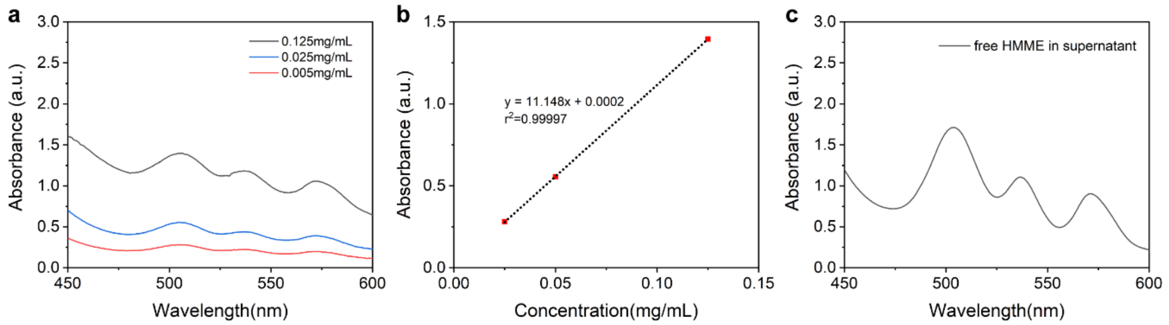

Figure S7. a-b, UV-Vis absorption curves (a) and corresponding standard curve at 505 nm absorption (b) of HMME at different concentrations. **c**, UV-Vis absorption curve of free HMME in the supernatant during a typical synthesis of HMME@BiL cells.

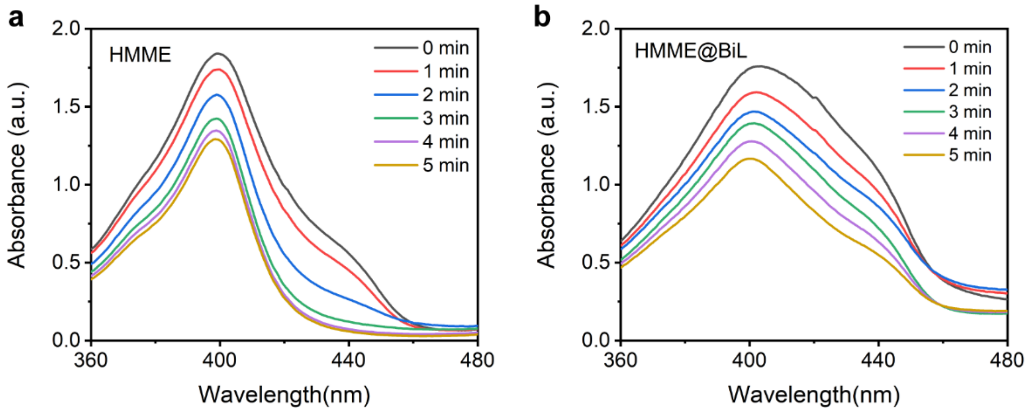

Figure S8. UV-vis spectra of DPBF based on HMME (a) and HMME@BiL (b) under US irradiation (1.0 MHz, 1.5 W/cm^2, 50% duty cycle) for varied durations.

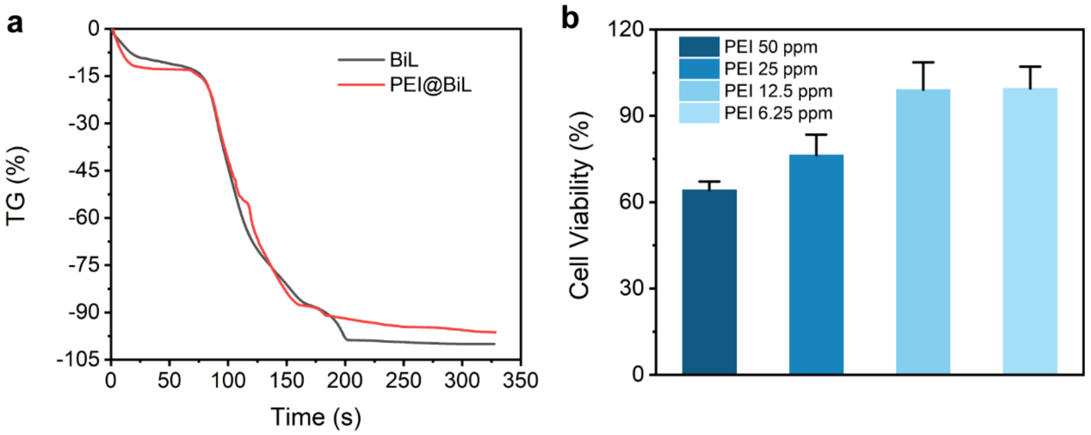

Figure S9. a,TG curve of BiL and PEI@BiL. **b,** The biocompatibility of the PEI in MRC-5 cells. Data were presented as mean ± s.d. n = 6.

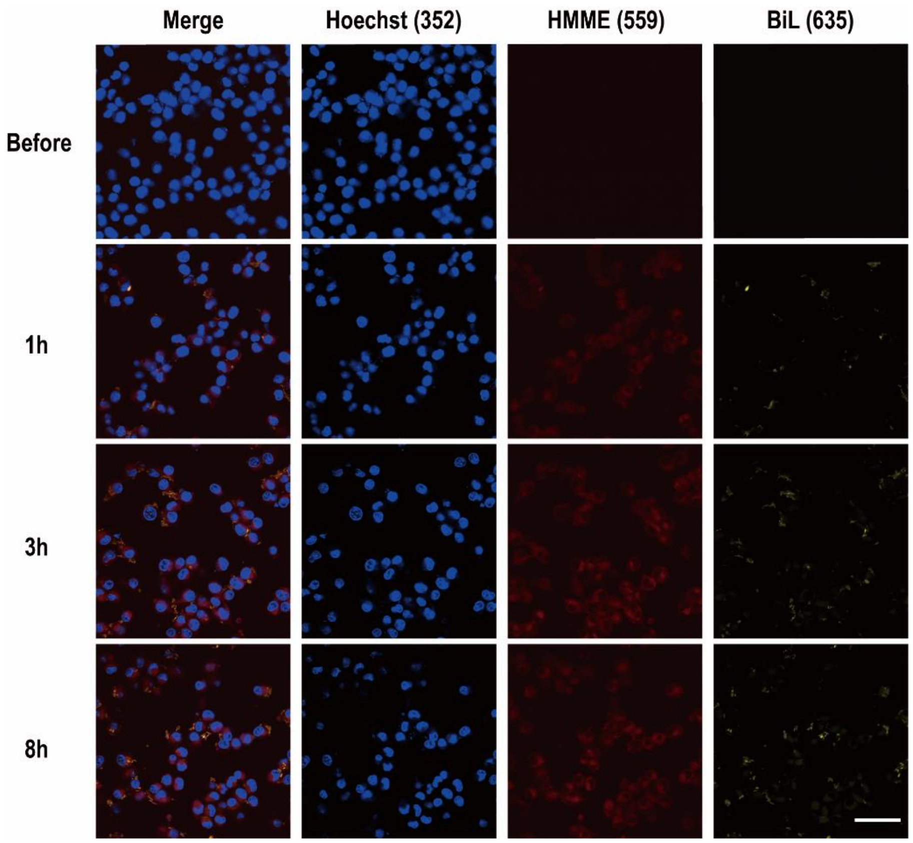

Figure S10. The fluorescent microscopic images of CT26 cells co-incubated with HMME@BiL (scale bar = 50 μm).

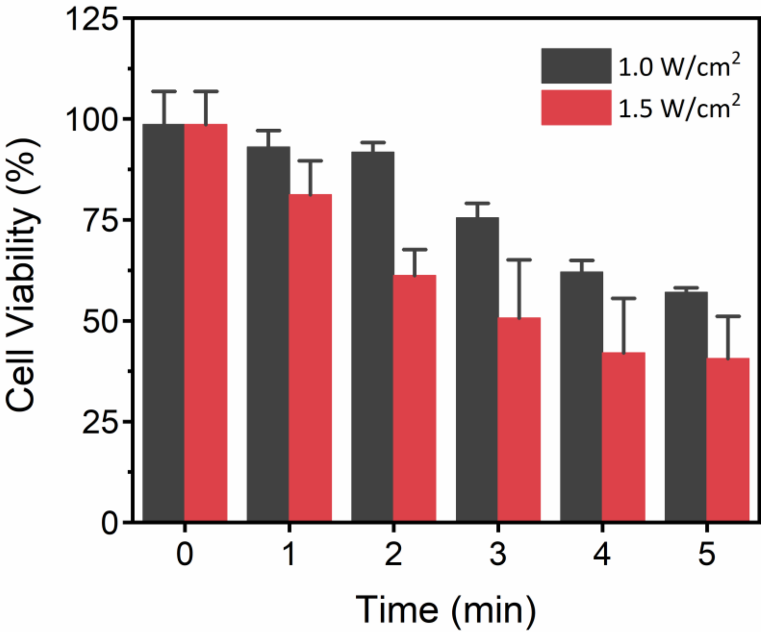

Figure S11. Relative cell viability of CT26 cells incubated with HMME@BiL under US irradiation with varied power densities durations. Data were presented as mean ± s.d. n = 6.

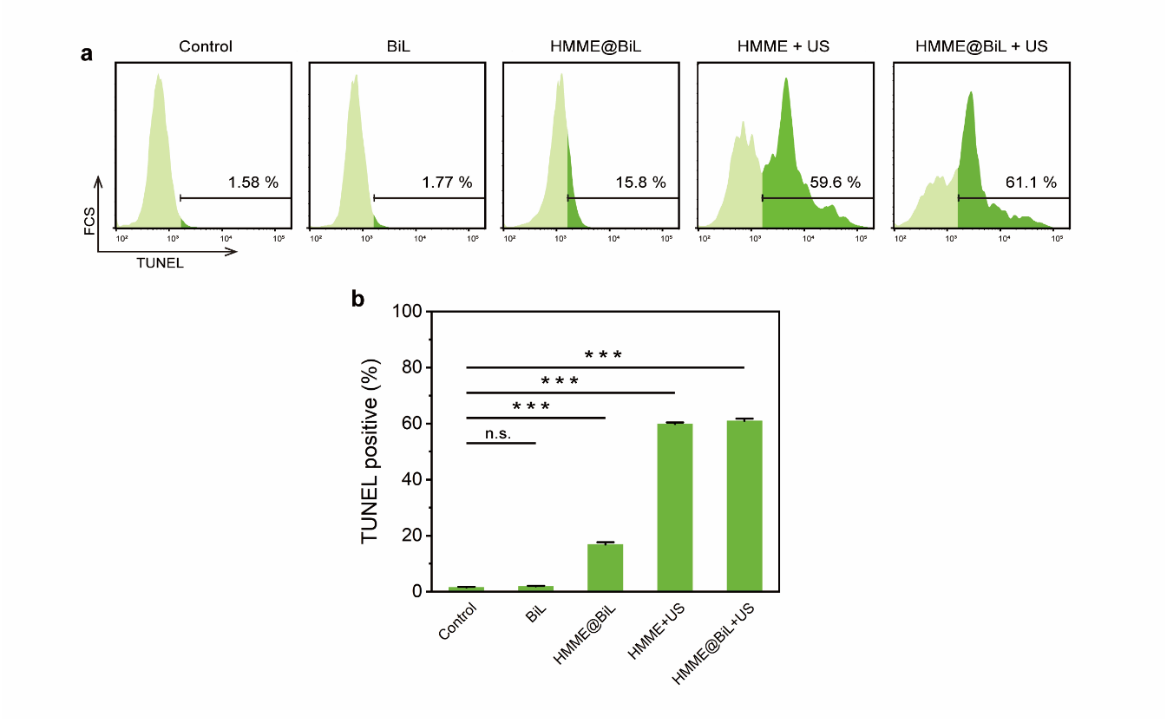

Figure S12. a, Flow cytometry analysis of CT26 cells stained with FITC-labeled TUNEL after various treatments: control (without any treatment), BiL only, HMME@BiL only, HMME + US and HMME@BiL + US. **b**, Quantification of the level of TUNEL positive CT26 cells. Data were presented as mean ± s.d. n = 3. Statistical significances were calculated via Student's t test, ***p<0.001 and n.s. for non-significant.

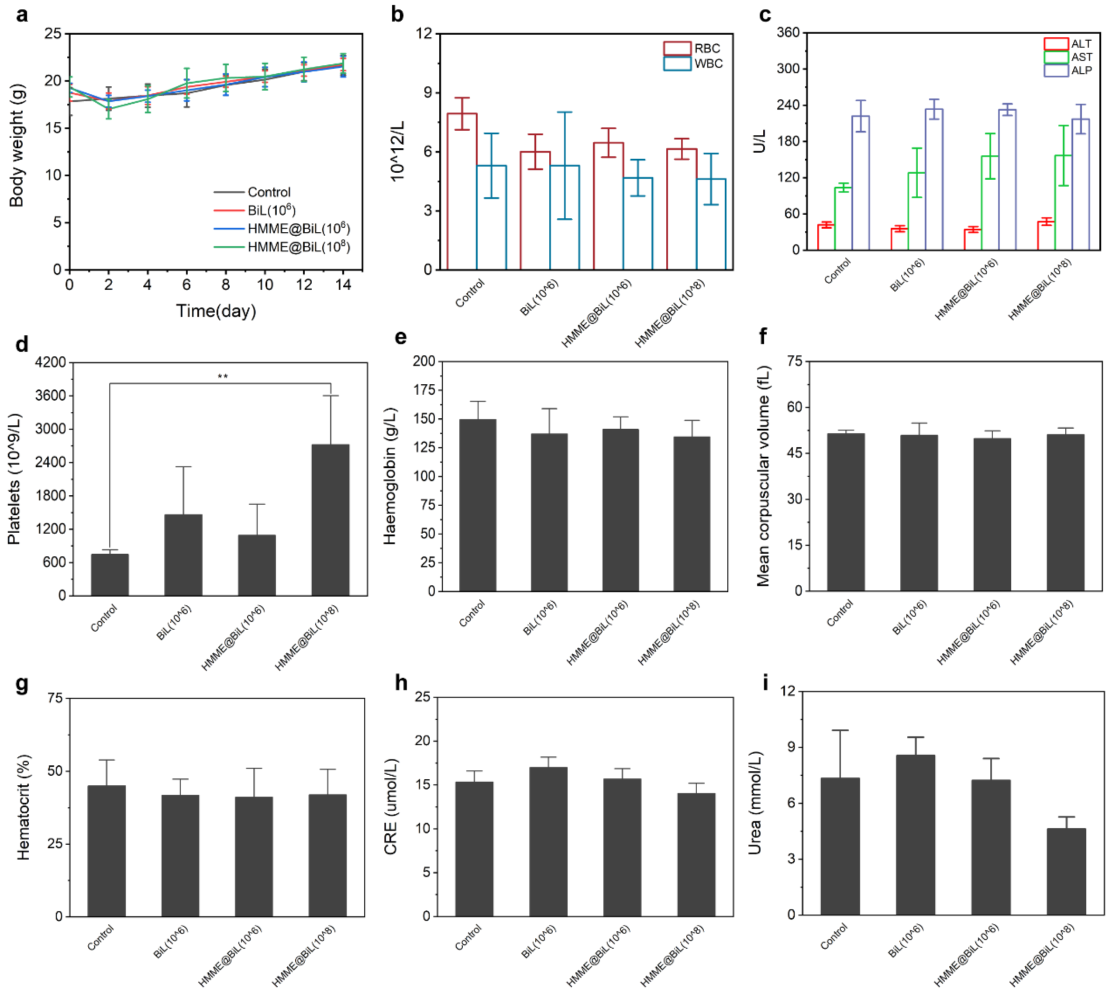

Figure S13. **a**, Bodyweight profile of mice during the *in vivo* biocompatibility evaluation. Data were presented as mean ± s.d. n = 5. **b-i**, Plasma biochemical and routine indexes of mice from different groups at the end of the *in vivo* biocompatibility evaluation. Data were presented as mean ± s.d. n = 4. Statistical significances were calculated via Student's t test, $**p<0.01$.

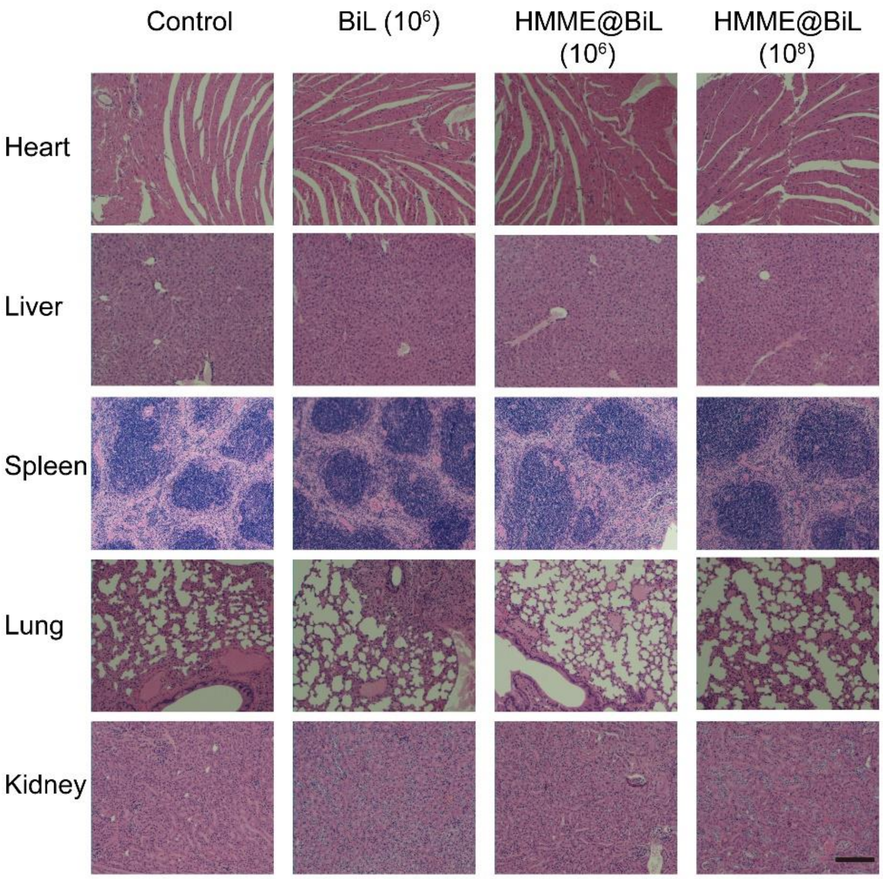

Figure S14. Microscopic images of the main organs of of mice during the *in vivo* biocompatibility evaluation (scale bar = 100 μm).

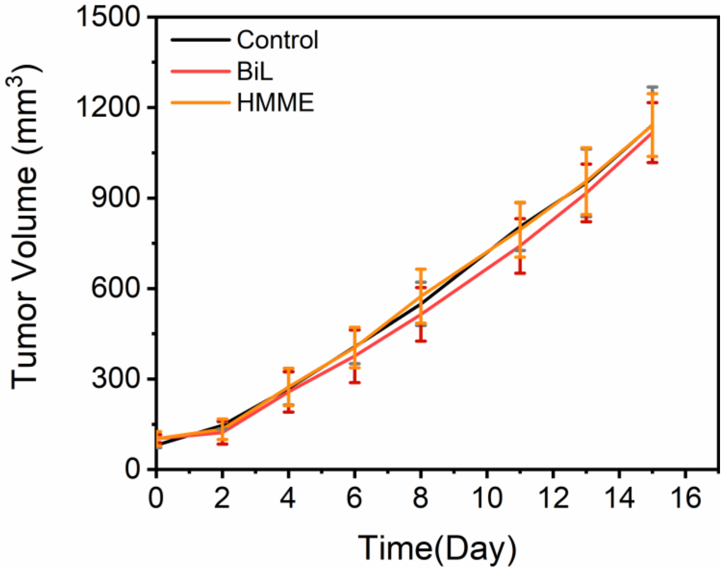

Figure S15. Tumor growth curves of mice from different groups after various treatments as indicated. Data were presented as mean ± s.d. n = 5.

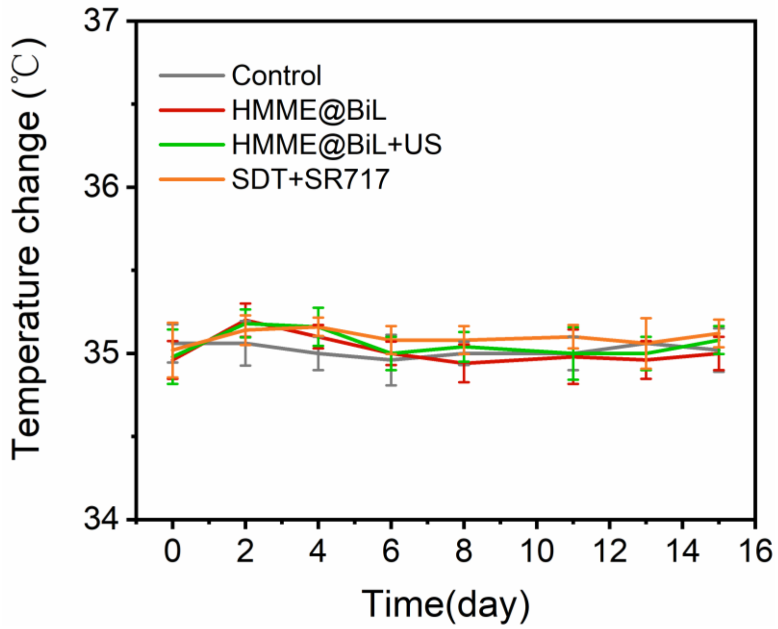

Figure S16. Temperature change of mice during the *in vivo* anti-tumor evaluation. Data were presented as mean ± s.d. n = 5.

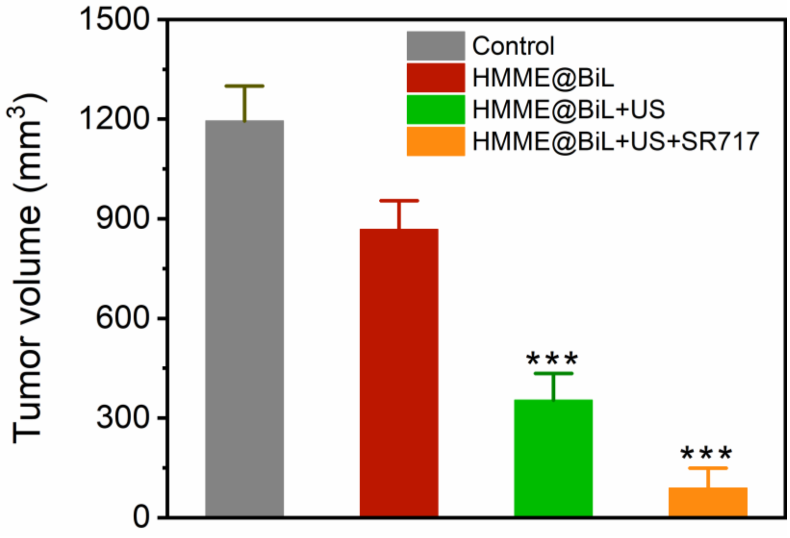

Figure S17. Average tumor volume profile of mice from different groups at the end of *in vivo* anti-tumor evaluation (unilateral model). Data were presented as mean ± s.d. n = 5. Statistical significances were calculated via Student's t test, ***p<0.001.

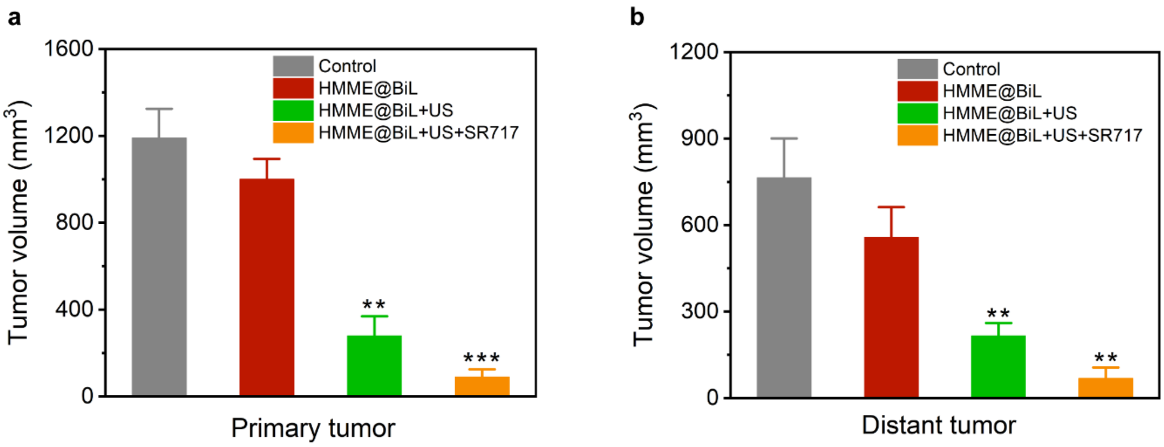

Figure S18. a-b, Average tumor volume profile of mice from different groups at the end of *in vivo* anti-tumor evaluation (bilateral model): primary tumor (a) and distant tumor (b). Data were presented as mean ± s.d. n = 5. Statistical significances were calculated via Student's t test, ***p<0.001 and ***p*<0.01.

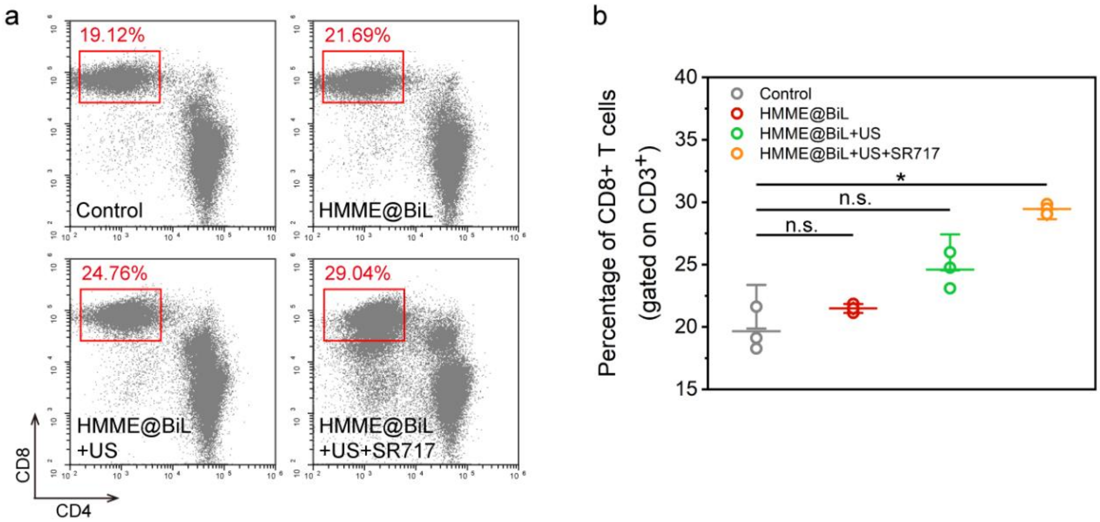

Figure S19. Flow cytometric results (**a**) and the corresponding statistical results (**b**) of $CD8^+$ expression gated on $CD3^+$ T cells isolated from spleens of CT26 bearing mice. Data were presented as mean ± s.d. n = 3. Statistical significances were calculated via Student's t test, **p*<0.05 and n.s. for non-significant.

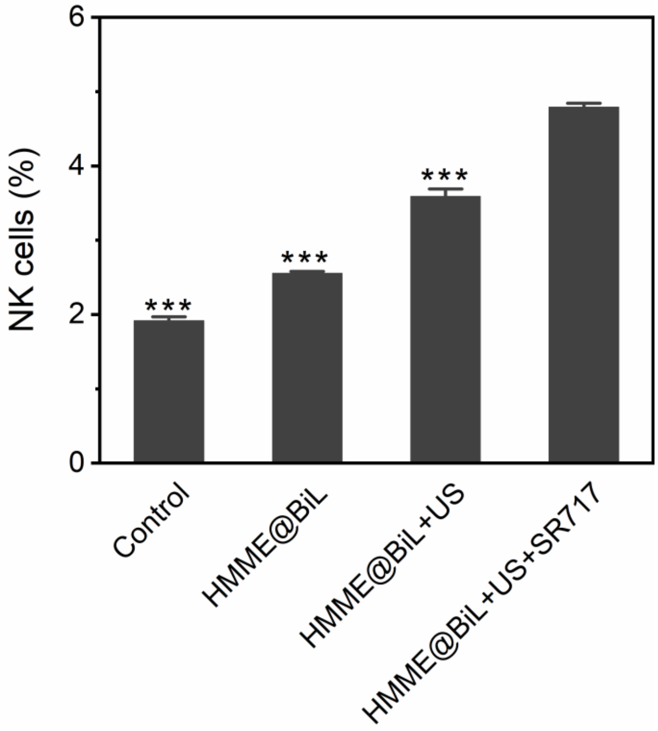

Figure S20. Percentage population of NK cells in the lymphocytes extracted from the spleen after indicated treatments. Data were presented as mean ± s.d. n = 3. Statistical significances were calculated via Student's t test, ***p<0.001.
